# Asymmetric BMP4 signalling improves the realism of kidney organoids

**DOI:** 10.1038/s41598-017-14809-8

**Published:** 2017-11-01

**Authors:** Christopher G. Mills, Melanie L. Lawrence, David A. D. Munro, Mona Elhendawi, John J. Mullins, Jamie A. Davies

**Affiliations:** 1 Deanery of Biomedical Science, University of Edinburgh, Edinburgh, EH8 9XB UK; 2 Centre for Cardiovascular Science, University of Edinburgh, Edinburgh, EH16 4TJ UK; 3 Clinical Pathology Department, Faculty of Medicine, Mansoura University, El-Mansoura, Egypt

## Abstract

We present a strategy for increasing the anatomical realism of organoids by applying asymmetric cues to mimic spatial information that is present in natural embryonic development, and demonstrate it using mouse kidney organoids. Existing methods for making kidney organoids in mice yield developing nephrons arranged around a symmetrical collecting duct tree that has no ureter. We use transplant experiments to demonstrate plasticity in the fate choice between collecting duct and ureter, and show that an environment rich in BMP4 promotes differentiation of early collecting ducts into uroplakin-positive, unbranched, ureter-like epithelial tubules. Further, we show that application of BMP4-releasing beads in one place in an organoid can break the symmetry of the system, causing a nearby collecting duct to develop into a uroplakin-positive, broad, unbranched, ureter-like ‘trunk’ from one end of which true collecting duct branches radiate and induce nephron development in an arrangement similar to natural kidneys. The idea of using local symmetry-breaking cues to improve the realism of organoids may have applications to organoid systems other than the kidney.

## Introduction

Here we present a strategy for increasing the anatomical realism of organoids by applying asymmetric cues that mimic spatial information present in embryonic development. We demonstrate the strategy by constructing kidney organoids of unprecedented realism in which nephrons are arranged around a collecting duct tree leading to a ureter-like trunk. The idea may be adaptable to a range of other organoid systems in developmental and regenerative research.

There is intense interest in producing kidney organoids to illuminate mechanisms of basic development^1^, to detect nephrotoxicants^2,3^ and to produce kidneys for renal replacement therapy^4–6^. The most basic type of organoid is produced when a suspension of progenitor cells is aggregated in culture. Using either mixed ex-fetu mouse nephrogenic, uretogenic and stromogenic cells^7^, or human iPS cells differentiated towards a renal fate^2,6^, the resulting organoid contains multiple, disconnected, immature collecting ducts, multiple immature nephrons, and stromal cells. The micro-anatomy is realistic but macroscopic organization is absent. More advanced, multi-stage techniques developed for mouse cells produce better macroscopic anatomy, producing nephrons in a distinct cortex connected to a single collecting duct tree radiating from the medulla, and loops of Henle dipping from the cortex into that medulla^8,9^. Nephrons in these cultures show physiological activity^10^ but a significant unrealistic feature remains: there is no ureter so the collecting duct tree has no exit.

In normal development, the ureter outside the kidney, and the collecting ducts within it, share a common origin, the ureteric bud, which evaginates from the Wolffian duct and enters the kidney as a coherent epithelial tubule^11^. Within the presumptive kidney, the bud causes metanephrogenic mesenchyme to arrange itself around the bud tips^12^ as ‘cap mesenchymes’^13,14^ and mesenchyme-derived signals cause the bud to branch to make a collecting duct system^15^. The ureteric bud left outside the kidney becomes ureter. There is evidence that fate choice between collecting duct and ureter is under mesenchymal control: the presumptive ureter becomes collecting duct if surrounded by metanephrogenic mesenchyme^16^, and treatment of whole kidney rudiments with bone morphogenetic protein 4 (BMP4), produced in peri-Wolffian mesenchyme but not in the metanephrogenic mesenchyme, causes the inner branches of the collecting duct system to express ureter-type markers^17^.

We have therefore tested, and confirmed, the hypothesis that imposition of an asymmetrical environment of BMP4 signalling onto a renal organoid will cause one branch of the collecting duct tree to take a ureter fate while the other branches develop as collecting ducts. As well as providing a solution to the specific problem of engineering renal organoids that have a ureter, the results provide a good illustration of the general approach of providing selected asymmetric cues to increase the realism of organoids.

## Materials and Methods

### Animals

No living animals were used in these experiments. All tissue used in this study was isolated from embryonic mice obtained from healthy CD1 adults which were culled, by methods listed under Schedule 1 of the UK Animals Scientific Procedures Act 1986, by trained staff licenced by the UK Home Office. All experiments were approved by the University of Edinburgh and performed in accordance with the institutional guidelines and regulations.

### Organ culture

Metanephric kidneys were isolated from E11.5 CD1 mouse embryos and, except in transplantation experiments, the Wolffian duct was removed. Kidney rudiments were cultured on 24 mm, 0.4 µm-pore membranes (Transwells, Corning 3450) in kidney culture medium (KCM: MEM [Sigma M5650], with 10% FBS, 1% penicillin/streptomycin). Ganeva-type organoids^8^ were made by incubating 8 embryonic kidneys in trypsin/EDTA (0.5 g/L trypsin:0.2 g/L EDTA, Sigma T4174), 37 °C, 2 min, rinsing them in KCM and dissociating them by trituration using a 200 μl tip and passage through a cell strainer (40 μm, BD Falcon). A pellet of these cells (3 mins, 8000 × g) was cultured overnight in 1.25 μM glycyl-H1152 dihyrochloride (ROCK inhibitor) in KCM on Isopore filters at the medium/gas interface (Trowell culture). Next morning, fresh KCM was applied for 4 h, 37 °C, 5% CO_2_, to produce ‘Unbekandt organoids’^7^. A single collecting duct progenitor epithelium was isolated from Unbekandt reaggregates and surrounded by mesenchyme isolated from 9–10 E11.5 kidneys, disaggregated and reaggregated as above, to produce Ganeva cultures. These were cultured 24 hr in Trowell culture before transfer to polyester membrane inserts (Transwells, Corning 3450). Grobstein cultures^18^ were made similarly but using an isolated ureteric bud tip of an E11.5 kidney instead of a ureteric bud sphere/tubule from a primary reaggregate.

### Tubule transplantation

Embryonic kidneys were cultured overnight in KCM containing rhodamine-conjugated peanut agglutinin (20 μg/ml, Vector Labs RL-1072). Labelled ureteric bud (UB) tubules were isolated manually, transplanted into either the metanephric- or the peri-Wolffian mesenchyme of a host E11.5 embryonic kidney, and cultured for 5 days on Transwell inserts (Corning 3450).

### BMP4 treatment

E11.5 mouse metanephric kidneys were cultured as above for 24hrs prior to BMP4 addition to the medium (5020-BP-010; R and D systems, reconstituted in 0.1% BSA (Bovine serum albumin), in PBS). BMP4 (concentrations used are described in the relevant results section) treatment was repeated daily until the end of the experiment (5 days).

### Bead Treatment

Protein loaded beads were prepared as follows: 5 Affi-gel beads (152–7302, Bio-Rad) were washed twice with PBS then incubated in 40 μl of BMP4 (5 μg/ml BMP4, 0.05% BSA, PBS), Gremlin (50 µg/ml, PBS), BSA (0.05%, PBS) or PBS for 1hr at room temperature, and rinsed in PBS. Gremlin beads were placed on embryonic kidneys that had been cultured for 24 hrs and beads were replaced daily until the end of the experiment. For experiments on natural kidneys, BMP4-soaked beads were also added to kidneys cultured for 24 hrs but replacement was found not to be necessary. BMP4-soaked beads were added to Grobstein kidneys (see above) once branching was observed, which was 24 hrs after being placed on polyester membranes. For Ganeva kidneys, BMP4-soaked beads were replaced daily for up to 4 days. In all those experiments involving beads, samples were cultured for 5 days after bead addition.

### Immunofluorescence

Samples were fixed in methanol at −20 °C, allowed to warm towards room temperature over 30 mins, washed in PBS, blocked in 5% BSA in PBS for 1hr and incubated in primary antibodies (Table S1) in 5% BSA overnight at 4 °C. They were washed in PBS, incubated with secondary antibodies (Table S2) in 5% BSA for 2hrs, washed 2x with PBS, mounted in H-1000 (Vecta shield) and imaged using a Zeiss Axiovert fluorescence microscope (Axiovision software) or a Nikon A1R confocal microscope (NIS elements software).

### Statistics

For categorical (feature present/ absent) data, 95% confidence intervals (CI^95%^) were calculated as ±1.96√(p(1 − p)/n) + 1/2n^19^. Hypothesis testing for these data was performed using N-1 Chi Squared tests^20^. All counts were generated by scoring absence/ presence of uroplakin expression, or branching, depending on the experiment.

### Data availability

The datasets can be found at: http://datashare.is.ed.ac.uk/handle/10283/2343.

## Results and Discussion

### Developing collecting ducts possess the plasticity required for conversion into ureter-type epithelium

The strategy of producing a ureter-like exit in a kidney organoid by converting one collecting duct branch into ureter-type epithelium depends on plasticity of the developing collecting duct. We and others have published evidence that the presumptive ureter can be induced to switch to a branching, nephron-inducing, collecting duct state by transferring it from the peri-Wolffian mesenchyme that normally surrounds it to metanephrogenic mesenchyme^16,21^. Can the opposite switch, from collecting duct to ureter, also be induced by changing mesenchymal environment? To test this, we performed a series of transplantation experiments in which sections of immature ureter or collecting duct epithelium, labelled with a fluorescent lectin for identification, were transplanted to the opposite type of mesenchyme.

**Figure 1:**
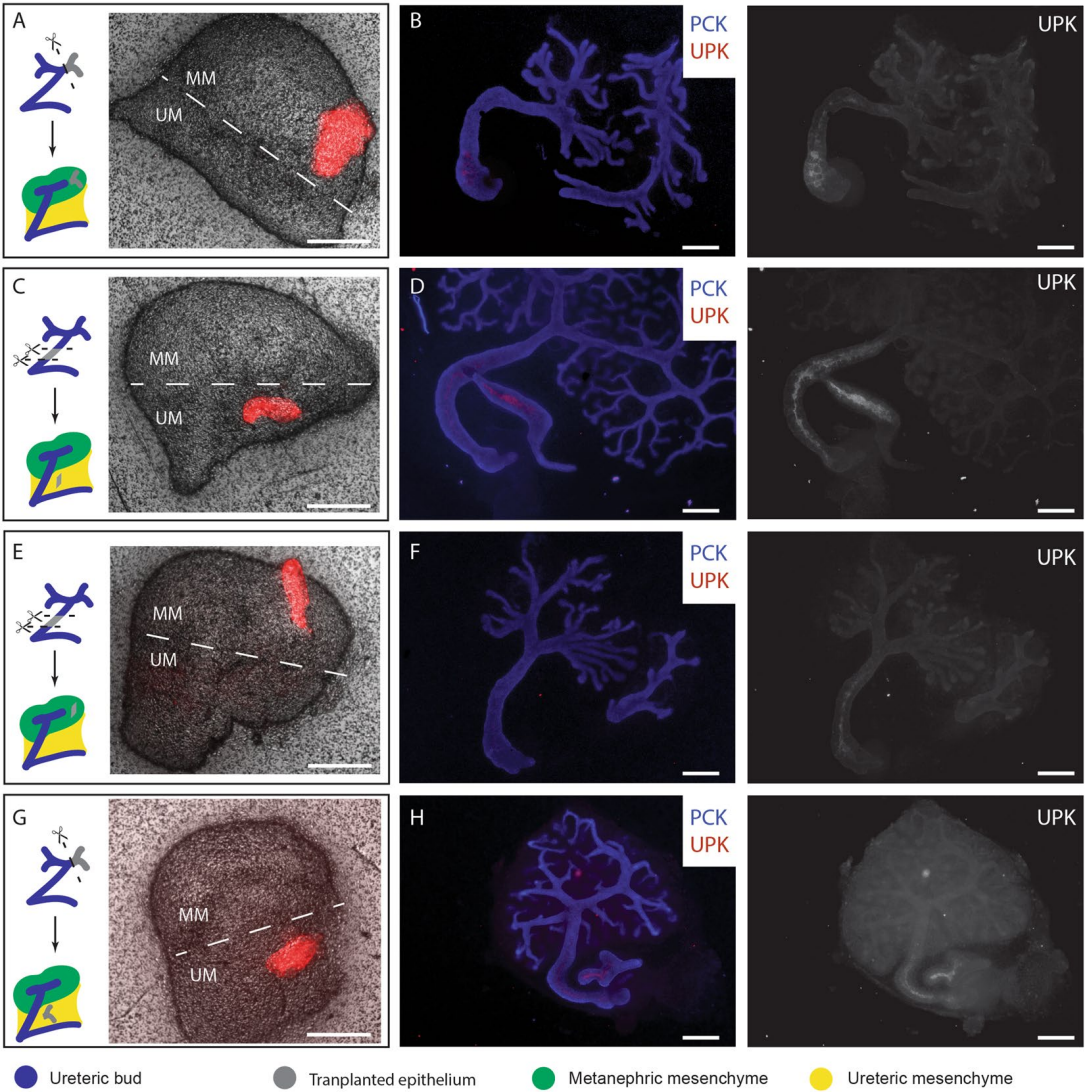
Renal mesenchyme dictates the identity of developing ureteric epithelium. Epithelia from the ureteric branches (**A** and **G**) or the ureteric stalk (**C** and **E**) were labelled with Rhodamine-conjugated Peanut agglutinin, isolated and transplanted into the metanephric mesenchyme (**A**,**B** and **E**,**F**) or the ureteric mesenchyme (**C**,**D** and **G**,**H**) of E11.5 kidneys. (**A**,**C**,**E**,**G)** show the position of transplanted epithelium (Red), with the approximate division between metanephric mesenchyme (MM) and prospective ureter mesenchyme (UM) annotated. (**B**,**D**,**F** and **H)** show development 5 days post transplantation: only grafts to peri-Wolffian mesenchyme become uroplakin-positive. UPK, Uroplakin. PCK, Pan-cytokeratin. Scale = 200 µm.

First, we performed control transplantations: grafting a section of E11.5 + 24 hr presumptive collecting duct into the metanephrogenic mesenchyme of a host E11.5 kidney resulted in its forming a branching tree (3/3 branched; 100%. CI^95%^ ± 17%) that did not express the ureter marker uroplakin (0/3 expressed uroplakin; 0%, CI^95%^ ± 17%) (Fig. 1A,B). Grafting presumptive E11.5 + 24 hr ureter from one kidney into the peri-Wolffian mesenchyme of a host resulted in it remaining unbranched (0/3 branched) and expressing uroplakin (3/3 expressed uroplakin, 100%. CI^95%^ ± 17%: Fig. 1C,D) demonstrating that the mere mechanics of grafting did not change fate. Grafting E11.5 + 24 hr presumptive ureter stalk into host metanephrogenic mesenchyme caused it to branch (5/5 branched 100%, CI^95%^ ± 10%) and to refrain from expressing uroplakin (4/5 did not express uroplakin; 80%, 95% CI^95%^ ± 39%: Fig. 1E,F), confirming (p = 0.040 by N-1 Chi-Square test) plasticity in the ureter-to-collecting duct direction. Critically, grafting a section of E11.5 + 24 h presumptive collecting duct into peri-Wolffian mesenchyme resulted in it remaining unbranched (0/2 branched; 0%, CI^95%^ ± 25%) and activating uroplakin expression (2/2 expressed uroplakin; 100%, CI^95%^ ±  = 25%, Fig. 1G,H). Reciprocal plasticity of the developing renal epithelium was quantified as those numbers of ureteric stalks that showed the collecting duct-like behavior of branching and those numbers of collecting ducts that showed the ureter-like behavior of expressing uroplakin. Reciprocal plasticity was not detected in renal epithelia transplanted into their natural mesenchyme (0/6: 0%, CI^95%^ ± 8.3%) but was in those transplanted into their unnatural mesenchyme (6/7; 85%, CI^95%^ ± 28.8%). This difference is significant (p = 0.003 by N-1 Chi-Square test).

### BMP4 can substitute for peri-Wolffian mesenchyme to induce uroplakin expression in presumptive collecting ducts

The results described above suggest that the development of distinct ureter and collecting duct from the same progenitor tissue might therefore arise simply from the asymmetry of mesenchymal influences: in the kidney, the bud is surrounded by metanephric mesenchyme and becomes collecting duct but the embryonic origins of the bud mean that one section of it crosses peri-Wolffian mesenchyme and so experiences a unique environment. If it were possible to identify the critical signal in the peri-Wolffian mesenchyme, it may be possible to apply it asymmetrically to an organoid and convert one collecting duct to ureter.

**Figure 2:**
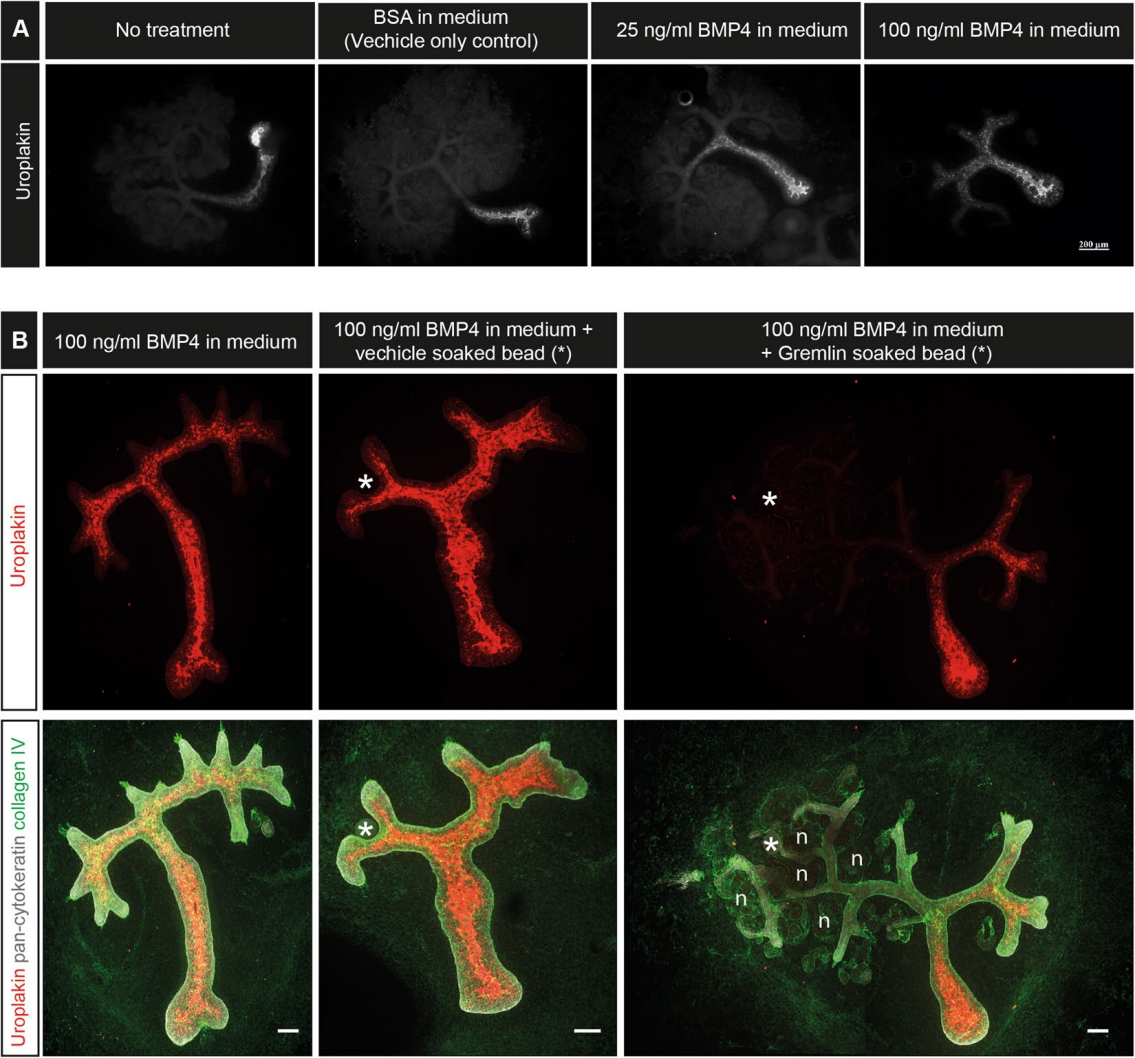
BMP4 induces expression of uroplakin in the developing collecting ducts. Developing collecting ducts were treated globally with BMP4 and uroplakin expression was investigated after 5 days of culture. (**A**) E11.5 kidney rudiments were cultured in media containing vehicle only (BSA), 25 ng/ml of BMP4 or 100ng/ml of BMP4. (**B**) Globally BMP4-treated E11.5 kidney rudiments were cultured in the presence of a Gremlin soaked bead or vehicle only soaked bead. *Bead location, n = a few examples of developing nephrons. Scale (unless otherwise stated) = 100 µM.

Peri-Wolffian mesenchyme secretes BMP4^22^ and culture of kidney rudiments in medium with high concentrations of BMP4 causes an expansion of the domain of uroplakin expression from the ureter, proximally, into the larger collecting ducts^17^. We confirmed this result (Fig. 2A). In kidneys cultured in standard medium or in medium supplemented with BSA, uroplakin expression was restricted to the ureter and did not extend to the first branch point of the collecting duct. In 25 ng/ml BMP4, uroplakin expression included the ureter and the primary branches of the collecting duct while in 100 ng/ml BMP4, expression extended even into secondary branches. To confirm that the uroplakin-inducing activity was indeed acting via BMP signalling (and not, for example, by an unknown contaminant in the BMP solution) we repeated the above experiments using 100 ng/ml BMP4 in the medium but placed an Affi-Gel bead soaked in either PBS or 50 µg/ml Gremlin, an inhibitor of BMP4^23^, at one side of the kidney rudiment. PBS-soaked beads had no effect on the BMP4’s ability to induce uroplakin expression and most or all of the branching ureteric buds expressed uroplakin (Fig. 2B, 5/5: 0%, CI^95%^ ± 10%, showed uroplakin expression near a PBS-soaked bead). Gremlin-soaked beads, on the other hand, protected nearby collecting ducts from BMP4-mediated induction of uroplakin (0/5, 0%, CI^95%^ ± 10%, showed uroplakin expression local to the Gremlin-soaked bead). Ducts distant from the bead still responded to BMP4. In addition, Gremlin restored nephron development, repressed by BMP4, in the area around the bead releasing it. Rescue by Gremlin indicates that BMP signalling does indeed mediate the uroplakin-inducing activity of medium supplemented by BMP4.

**Figure 3:**
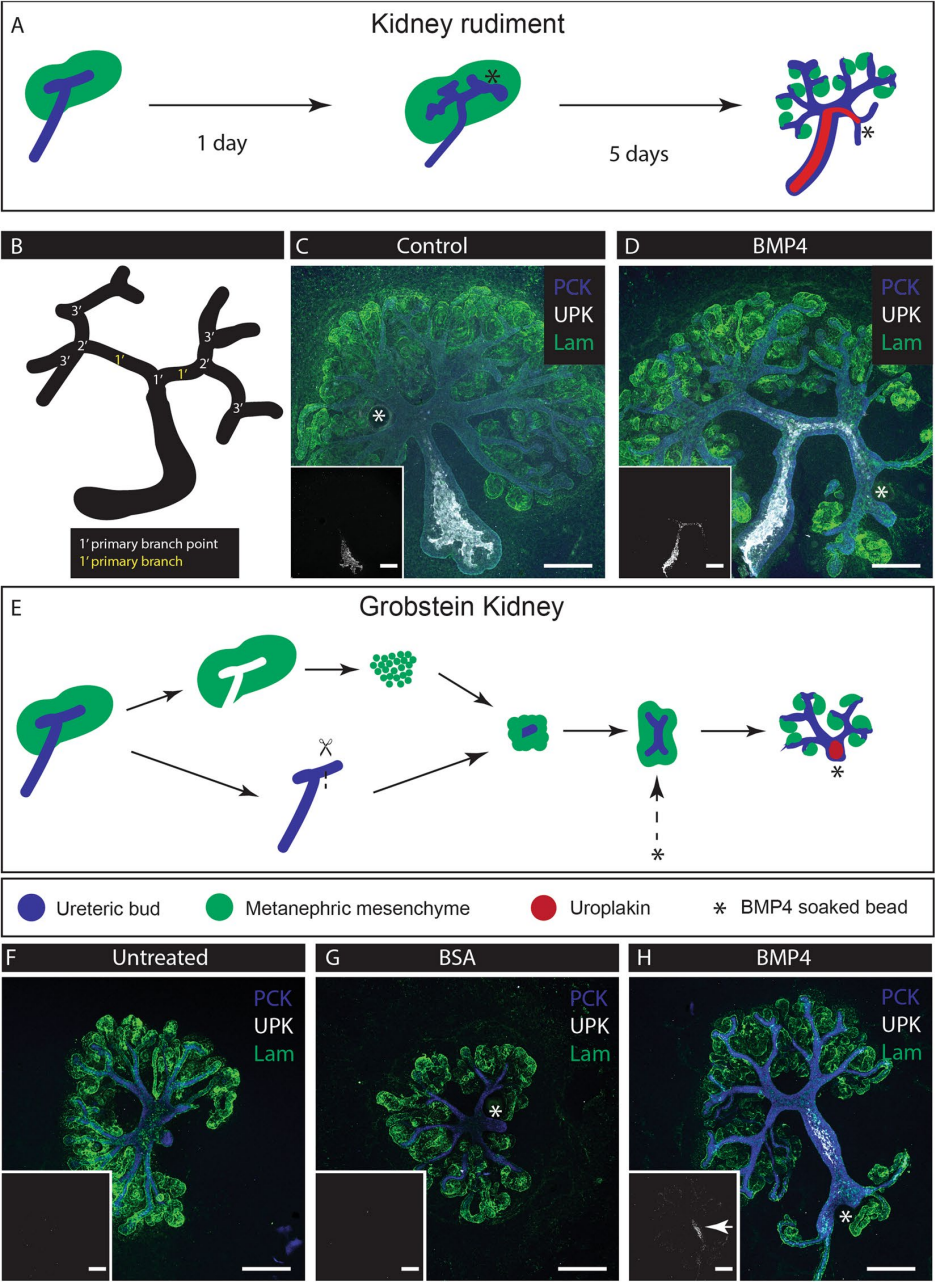
Local administration of BMP4 induces expression of Uroplakin in targeted developing collecting duct. Developing collecting ducts were exposed to BMP4 beads. (**A**) Illustration of the treatment method (*Bead). (**B**) Nomenclature of branches. (**C**) and (**D)** E11.5 kidneys cultured for 24hrs then given a bead soaked in either BSA (Vehicle: C) or BMP4 (**D**) at a secondary branch point. (**E)** Application of the beads to Grobstein culture; (**F**) Untreated; (**G**) BSA-treated; (**H**) BMP4 treated. Inserts: isolated uroplakin channel. UPK, Uroplakin. PCK, Pan-cytokeratin. Lam, Laminin. Arrows indicate induced uroplakin expression. Scale bar = 200 µm.

Miyazaki *et al*. applied BMP4-soaked Affi-Gel beads to kidneys and reported local inhibition of nephron development but did not assess effects on collecting duct/ ureter differentiation^24^. To explore the possibility of driving local ureter-type differentiation, we treated intact cultured E11.5 + 24 h kidney rudiments with a BMP4-soaked Affi-Gel bead located near one specific secondary ureteric bud branch point (Fig. 3A and B), and observed the behaviour and uroplakin expression of nearby and distant collecting ducts. Control beads carrying only BSA had no effect on branching, nephrogenesis or uroplakin expression (Fig. 3C, 0/14 expressed uroplakin; 0%, CI^95%^ ± 3.6%). BMP4-soaked beads reduced branching and nephrogenesis locally and drove an expansion of uroplakin expression from the ureter into the collecting duct near the bead (Fig. 3D, 13/13: 100%, CI^95%^ ± 3.8%).

This expansion of the uroplakin-expressing domain might reflect ureter-type differentiation of collecting duct cells, or it might be caused by beadward migration of already uroplakin-positive cells from the ureter stalk. To discriminate between these possibilities, we used a culture method from Grobstein^18^, isolating E11.5 ureteric tips which do not express uroplakin and surrounding them with metanephrogenic mesenchyme (Fig. 3D). These cultures formed an anatomy like that of Ganeva-type renal organoids (see below), in that they contained a collecting duct system with no uroplakin-positive cells (Fig. 3F). Treatment of such kidneys with a control bead did not affect branching or nephron formation and did not induce uroplakin (Fig. 3G, 0/3 expressed uroplakin: 0%, CI^95%^ ± 17%). Application of a BMP4-soaked bead to such kidneys induced a nearby collecting duct to grow straight and to express uroplakin (Fig. 3H, 5/5 expressed uroplakin: 100%, CI^95%^ ± 10%). A N-1 Chi Square test between the control and BMP4 bead-treated populations yields p = 0.0082, allowing us to reject the hypothesis that BMP4 fails to induce uroplakin expression in cells derived from bud type. What is more, this tubule was nephron-free along its uroplakin positive segment. This suggests that BMP4 induces a ureter-type differentiation in immature collecting duct, and that BMP4- soaked beads are a viable method of generating an asymmetric environment that can cue local ureter formation.

### BMP4-soaked beads break the symmetry of Ganeva-type renal organoids and produce a ureter-like trunk for the collecting duct tree

The most anatomically realistic renal organoids are made using a method based on Ganeva *et al*.^8^, in turn based on Unbekandt *et al*.^7^. The Unbekandt method aggregates a suspension of renal progenitor cells, under temporary anti-apoptotic pharmacological support, and results in formation of many scattered epithelial spheres, tubules and small trees characteristic of primitive collecting duct and, shortly afterwards, many developing nephrons scattered among and connecting to them. The micro-anatomy of the system is realistic but the formation of many independent collecting duct tubules instead of one coherent tree means realistic large-scale anatomy is missing. If one presumptive collecting duct tubule is isolated from an Unbekandt-type organoid and combined with reaggregated or intact renal metanephrogenic mesenchyme, it produces a single, coherent tree that organizes realistic large-scale anatomy. Like Grobstein culture (Fig. 2F), these Ganeva-type organoids still lack a ureter because the collecting duct develops in a symmetric environment. This raises the possibility that imposition of an appropriately asymmetrical environment may be a way to persuade one end of the organoid to make a ureter (we use the term ‘asymmetry’ in the sense of making the environment of one end of an organoid different from that of the other).

**Figure 4:**
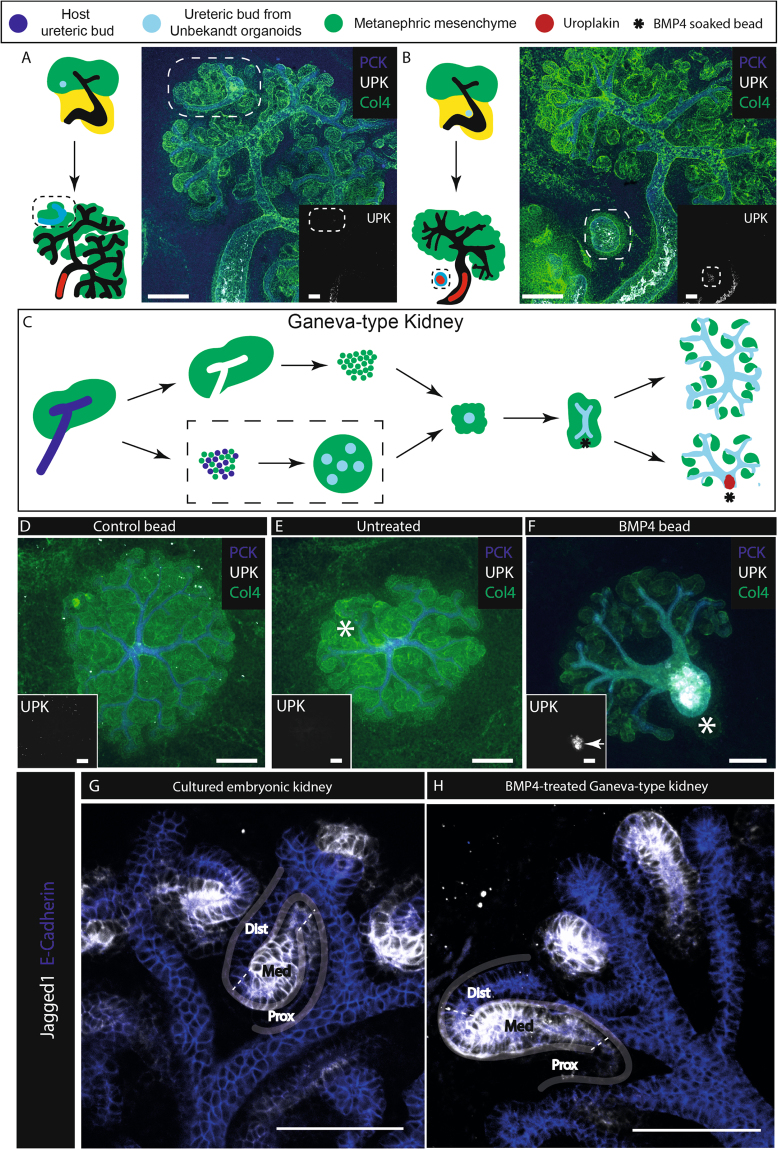
Unbekandt-culture-derived ureteric bud spheres respond to BMP4 to express uroplakin. (**A**) and (**B**) Ureteric bud structures (outlined by dashed lines), isolated from Unbekandt orgranoids^7^ were transplanted into host metanephric (**A**) or peri-Wolffian (**B**) mesenchyme of E11.5 kidneys and cultured for 5 days. Induction of uroplakin is seen when transplanted into peri-Wolffian mesenchyme. (**C**) Generation and bead treatment of Ganeva kidneys: ureteric spheres are obtained from 1-day Unbekandt organoids then isolated and surrounded by e11.5 metanephric mesenchyme to form Ganeva kidneys, which were treated 2 days later with BMP4 soaked beads changed daily for up to three days. The dashed box highlights differences between the Ganeva and Grobstein method. (**D**) Ganeva kidney with no treatment; (**E**) treatment with a BSA-soaked bead; (**F**) A BMP4-soaked bead induces the nearby tubule to become an unbranched, uroplakin-positive, nephron-free tubule. (**G**) Nephron patterning of natural cultured kidneys is comparable to BMP4-treated Ganeva kidneys (**H**). Outlines of developing nephrons has been drawn on and approximate sections annotated (Dist, Distal tubule; Med, Medial tubule; and Prox, Proximal tubule). UPK, Uroplakin. PCK, Pan-cytokeratin, Col4, Collagen Type IV. Arrows indicate induced uroplakin. Scale bar = 200 µm.

To determine whether BMP4-soaked Affi-Gel beads might be used to impose the necessary asymmetry, we first performed a small-scale experiment to test whether collecting duct progenitor epithelia isolated from Unbekandt-type organoids could be induced to express uroplakin. We did this by transplanting them into host cultured kidney rudiments. Grafted into the metanephric mesenchyme they formed small branching trees (2/2 branched; 100%, CI^95%^ ± 25%) that induced nephrons and did not express uroplakin (Fig. 4A, 0/2 expressed uroplakin; 0%, CI^95%^ ± 25%). Grafted into the peri-Wolffian mesenchyme of a host kidney, they did not branch (0/2 branched; 0%, CI^95%^ ± 25%) and did not induce nephrons, but they did express uroplakin (Fig. 4B, 2/2 expressed uroplakin; 100%, CI^95%^ ± 25%).

Having established that collecting duct progenitors isolated from Unbekandt-type renal organoids are capable of being induced to express uroplakin, we went on to test whether BMP4-soaked Affi-Gel beads could induce local ureter-type differentiation when collecting duct progenitors of this type are used as the basis for producing Ganeva-type organoids (Fig. 4C). Ganeva-type organoids that included no beads produced symmetrical cultures with nephrons around a collecting duct tree that had no ureter-type trunk (Fig. 4D, 0/8 expressed uroplakin or possessed a trunk; 0%, 95% CI^95%^ ± 6.3%). Those incorporating a control bead were similar (Fig. 4E, 0/3 expressed uroplakin or possessed a trunk; 0%, CI^95%^ ± 17%). Those treated with BMP4-soaked Affi-Gel beads, replaced daily for up to 3 days, showed a dramatically different anatomy. Away from the bead, collecting duct branching and nephron formation proceeded normally but, critically, the collecting duct near the bead did not branch or induce nephrons but instead grew thick and showed strong expression of uroplakin (Fig. 4F, 15/23 expressed uroplakin; 65%, CI^95%^ ± 28%). A comparison of cultures treated with BMP4 beads with pooled control cultures, using a N-1 Chi Squared test, allows us to reject the null hypothesis of no difference at p = 0.0004.

As a check that induction of uroplakin at one end of an organoid, by local BMP treatment, did not affect segmentation of nephrons produced at the other end of the culture, we stained them for Jagged1 and E-cadherin. Normally, Jagged1 is expressed in the medial segment of a maturing nephron^25,26^, while E-cadherin is expressed in the distal segment and the collecting duct^25^. We confirmed this in normal cultured kidneys (Fig. 4G). In organoids selected for those that showed BMP4-induced uroplakin expression, E-cadherin was still expressed in collecting ducts and distal tubules, and Jagged 1 was still expressed in medial segments (Fig. 4H). This suggests that BMP4-soaked beads do not disrupt nephron patterning.

We acknowledge that there is a difference between having a uroplakin-expressing tubule and having a fully-functional ureter, and much remains to be done to produce a properly structured urothelium with attendant smooth muscle etc. Nonetheless, here we have shown that the application of a local signal, designed to mimic an asymmetry present in the natural embryo but absent from self-organized organoid systems, can be used to produce a more realistic organoid with features that do not appear when self-organization alone is used. This idea could be applied to other renal organoids (for example, those produced from human iPS cells^3^) and to organoids made from other parts of the embryo and adult. The technique can be used both to explore the limits of self-organization, and to transcend them.

## Electronic supplementary material

Supplementary Information
