# Supplementary material for: Asymmetric BMP4 signalling improves the realism of kidney organoids
Source: Sci Rep. 2017 Nov 1;7:14824. doi: 10.1038/s41598-017-14809-8 (PMC5665994; doi:10.1038/s41598-017-14809-8)
Supplement: Supplementary file 1 — Supplementary Information [file 41598_2017_14809_MOESM1_ESM.doc]

**Asymmetric BMP4 signalling improves the realism of kidney organoids**

Christopher G. Mills*^1,2^, Melanie L. Lawrence^1^, David A.D. Munro^1^, Mona Elhendawi^1,3^, John J. Mullins^2^, Jamie A. Davies*^1^

^1^Deanery of Biomedical Science, University of Edinburgh, EH8 9XB, UK. ^2^ Centre for Cardiovascular Science, University of Edinburgh, Edinburgh, EH16 4TJ, UK. ^3^ Clinical Pathology Department, Faculty of Medicine, Mansoura University, El-Mansoura, Egypt.

*Correspondence to C.G.Mills@sms.ed.ac.uk, jamie.davies@ed.ac.uk

**SUPPLEMENTARY DATA**

**Table S1. List of Primary antibodies used**

| **Primary** | **Host Species** | **Company** | **Catalog number** | **Working dilution** |
| --- | --- | --- | --- | --- |
| Pan-cytokeratin | Mouse | Sigma | C2562 | 1/100 |
| Collagen IV | Goat | Millipore | AB769 | 1/100 |
| Uroplakin | Rabbit | Kindly gifted by Tung-Tien Sun Lab (New York University) |  | 1/500 |
| Jagged 1 | Goat | R and D systems | AF599 | 1/200 |
| E-Cadherin | Mouse | BD Transduction Labs | 610182 | 1/200 |
| Laminin | Chick | Abcam | ab14055 | 1/100 |

**Table S2. List of Secondary Antibodies used**

| **Secondary** | **Host** | **Company** | **Catalog number** | **Working dilution** | **Conjugate** |
| --- | --- | --- | --- | --- | --- |
| Anti-mouse | Horse | Vector Labs | CI-2000 | 1/100 | AMCA |
| Anti-Goat | Donkey | Invitrogen | A11055 | 1/200 | Alexa Fluor 488 |
| Anti-Rabbit | Donkey | Invitrogen | A21207 | 1/200 | Alexa Fluor 594 |
| Anti-chick | Goat | Abcam | 97134 | 1/200 | FITC |
| Anti-mouse | Donkey | Invitrogen | A31571 | 1/200 | Alexa Fluor 647 |
